# Determinants of the citation rate of medical research publications from a developing country

**DOI:** 10.1186/2193-1801-3-140

**Published:** 2014-03-14

**Authors:** Anupama Annalingam, Hasitha Damayanthi, Ranil Jayawardena, Priyanga Ranasinghe

**Affiliations:** Department of Pharmacology, Faculty of Medicine, University of Colombo, Colombo, Sri Lanka; Institute of Health and Biomedical Innovation, Queensland University of Technology, Brisbane, Queensland Australia

**Keywords:** Citation rate, Determinants, Medical research, Sri Lanka, Developing country

## Abstract

**Background:**

The number of citations received by an article is considered as an objective marker judging the importance and the quality of the research work. The present study aims to study the determinants of citations for research articles published by Sri Lankan authors.

**Methods:**

Papers were selectively retrieved from the SciVerse Scopus® (Elsevier Properties S.A, USA) database for 10 years from 1st January 1997 to 31st December 2006, of which 50% were selected for inclusion by simple random sampling. The primary outcome measure was citation rate (defined as the number of citations during the 2 subsequent years after publication). Citation data was collected using the SciVerse Scopus® Citation Analyzer and self citations were excluded. A linear regression analysis was performed with ‘number of citations’ as the continuous dependent variable and other independent variables.

**Result:**

The number of publications has steadily increased during the period of study. Over three quarter of papers were published in international journals. More than half of publications were research studies (55.3%), and most of the research studies were descriptive cross-sectional studies (27.1%). The mean number of citations within 2 years of publication was 1.7 and 52.1% of papers were not cited within the first two years of publication. The mean number of citations for collaborative studies (2.74) was significantly higher than that of non-collaborative studies (0.66). The mean number of citations did not significantly change depending on whether the publication had a positive result (2.08) or not (2.92) and was also not influenced by the presence (2.30) or absence (1.99) of the main study conclusion in the title of the article. In the linear regression model, the journal rank, number of authors, conducting the study abroad, being a research study or systematic review/meta-analysis and having regional and/or international collaboration all significantly increased the number of citations.

**Conclusion:**

The journal rank, number of authors, conducting the study abroad, being a research study or systematic review/meta-analysis and having regional and/or international collaboration all significantly increased the number of citations. However, the presence of a positive result in the study did not influence the citation rate.

## Background

Publication of an article in a peer-reviewed journal is the initial step in the dissemination of research findings through the scientific community, but continued dissemination depends on the citation of the original work in subsequent publications. The number of citations received by an article is viewed as an objective marker judging the importance and the quality of the original research work (Seglen 1997). Having a research article cited by multiple sources not only facilitates the dissemination of its message among the medical community, it also encourages its application to patient care (Willis et al. 2011). Objectively assessing the quality of research work and scientific writing is important in order to improve the standard of future scientific work. The “Impact Factor” of a journal which is also calculated based on citations received in a year for all articles published in the journal in the previous 2 years, is a quantitative measure of the quality of the journal, its articles and the authors (Amin and Mabe 2003). It is used to compare the relative impact between scientific journals.

Citation analysis deals with the frequencies and patterns of citations in journals and the various factors that determine the number of citations of journal articles. The number of peer-reviewed scientific publications in the medical literature has dramatically increased during the last few decades (Stringer et al. 2008). For example a total of 8.1 million journal articles were published in MEDLINE alone between 1978 and 2001 (Druss and Marcus 2005). It has previously been reported that more than half of all published scientific articles are never cited by any other paper (Hamilton 1991). This is further complicated by the rapidly increasing number of biomedical journals and research publications. According to available literature length of title, type of research work, journal prestige and publication bias are some of the factors associated with higher number of citations (Callaham et al. 2002; Jacques and Sebire 2010a). However, majority of these studies are from western developed countries and the factors determining citations/impact of research articles from developing countries have not been previously studied.

Sri Lanka, is a rapidly developing island nation in the Indian subcontinent that has a population of nearly 20.5 million (Department of Census and Statistics Sri Lanka 2010). Sri Lanka is recognized internationally for its good health indicators at a relatively low level of GDP (approx 2,500) and it is at the forefront in the South Asian region in providing quality health services (Sri Lanka Department of Health Service 2002). Scientific research is an essential component in guiding improvements in health systems and development of new initiatives (Commission on Health Research for Development 1990). Sri Lanka has contributed only 0.08% of the global research output in health and biomedical sciences during the last decade (from 2000–2009) (Ranasinghe et al. 2012). The Ceylon Medical Journal is the only medical journal from Sri Lanka indexed in PubMed, Web of Science, Scopus and other popular medical databases (Ranasinghe et al. 2011). The remaining national medical journals are mainly in circulation within the local scientific community (Ranasinghe et al. 2011).

The impact of Sri Lankan medical research is also relatively low, with over 30% of publications having no citations, a fact which is common to research from most developing countries (Ranasinghe et al. 2012). It is important to identify factors that contribute towards the lower number of citation/impact. At present there are no studies evaluating citations of biomedical research publications from Sri Lanka and other developing countries in the South Asian region. The present study aims to study the determinants of citations for research articles published by Sri Lankan authors.

## Methods

### Search strategy

This study was based on the Sri Lankan medical research publication data, retrieved from the SciVerse Scopus® (Elsevier Properties S.A, USA) database for 10 years from 1st January 1997 to 31st December 2006. The process used in article selection was as follows: affiliation–‘Sri Lanka’ or ‘Ceylon’, Publication year–‘1st January 1997’ to ‘31st December 2006’, Subject area–‘Life and Health Sciences’ and Language–‘English’. Conference proceedings, trade publications, books and book series were excluded.

The above search strategy identified 1,536 articles, of which 50% were selected for inclusion in the present analysis using the following method. Citations for the 1,536 articles were retrieved from SciVerse Scopus® database and articles were arranged in the descending order of citations. Subsequently they were categorized into groups of 10 in the same order and from each group of 10 articles, 5 were selected using simple random sampling using computer generated random numbers.

### Data extraction

Data were extracted from the included studies by perusal of abstract or full-text (when necessary) by one reviewer (AA) using a standardised form and checked for accuracy by a second reviewer (HD). The data extracted from each study were: a) study details–lead author, number of authors, country of study, year published, journal name and journal type (International or Local), b) methods–article type (research study, audit/editorial/letter, systematic review/meta-analysis, case report/case series), study design (case–control, descriptive cross-sectional, cohort, experimental, pre-clinical trial, clinical trial, other), and sample size, c) results–positive results and presence of study conclusion in title, d) number of citations and e) other–number of words in title, collaboration (local, regional, international) and number of collaborating countries. Discrepancies in the extracted data were resolved by discussion, with involvement of a third reviewer (PR) when necessary.

The primary outcome measure was citation rate. Since articles are rarely cited during the same year in which they were published, the citation rate was defined as the number of citations during the 2 subsequent years after publication. Citation data was collected using the SciVerse Scopus® Citation Analyzer and self citations were excluded to evaluate true impact. A self-citation was defined as a citation by any co-author of the original article. One member (RJ) of the investigative team performed the citation analysis and a second reviewer (PR) confirmed accuracy in a 10% random sample. To measure the influence of the journal on the number of citations the SCImago Journal Rank (SJR) was derived for each journal for the particular year in which each individual article was published from the SciVerse Scopus® database. SJR is a measure of scientific influence of scholarly journals that accounts for both the number of citations received by a journal and the importance or prestige of the journals where such citations come from (Falagas et al. 2008).

### Data analysis

We performed all analyses using SPSS statistical software version 14.0 (SPSS Inc, Chicago, Ill). As citation data were non-parametrically distribute the significance of the differences between proportions (%) and means was tested using the Z test and Mann–Whitney *U* test or Kruskal-Wallis H test respectively, and Fisher’s Least Significant Difference test was used for post hoc analysis. Citations under sub-categories (local/international journal, article type, study design, etc.) are described as mean values with range and/or 95% CI. Since majority of the articles were never cited medians were not used during analysis, as under most sub-categories the median was ‘0’ due to non-citation. A linear regression analysis was performed with ‘number of citations’ as the continuous dependent variable and number of authors (continuous), SJR (continuous), number of words in title (continuous), type of journal (binary), type of study (0 = case reports/case series), country of study (0 = local), presence of collaboration (binary) and type of collaboration (0 = local) as the independent variables. The explanatory independent variables that were associated with the dependent variable in univariate analysis (p < 0.25) were selected to be included in the regression analysis. The explanatory variables selected above were subsequently included in the linear regression model, a backward elimination procedure was used and a p-value of 0.10 was considered as the cut-off for removal of variables. For each independent variable with more than two categories dummy variables were created and the first category was taken as the reference category. In all analysis a P values of < 0.05 was considered statistically significant.

## Results

The total number of articles selected according to the above selection strategy was 768. The number of publications have steadily increased from 1997 (n = 55) to 2006 (n = 126). Majority of the articles were published in international journals (75.4%) (Table 1). However, of the individual journals, the Ceylon Medical Journal (n = 176, 22.9%) had the highest number of publication, followed by the British Medical Journal (n = 21, 2.7%) and the Lancet (n = 19, 2.5%). A significant majority of the publication were research studies (55.3%), and most of the research studies were descriptive cross-sectional studies (27.1%) (Table 1). The three main specialities studied were; Parasitology (n = 81, 10.5%), Psychology (n = 50, 6.5%) and Toxicology (n = 50, 6.5%). More than 80% of the studies were conducted solely in Sri Lanka, with 48.7% (n = 374) being collaborative studies with participation of multiple institutions in Sri Lanka or institutions from other countries (Table 1). Most of the inter-country collaborative studies were between Sri Lanka and one (n = 171, 66.0%) or two (n = 48, 18.5%) other countries. The main conclusion of the study was given in the title of the article in 16.5% (n = 127) studies. In addition 36.2% of the research studies (n = 278) had a positive result in relation to the evaluated outcome. The mean number of subjects in research studies and the mean number of authors were 577.7 (range 10–13986; 95% CI 405.9–749.6) and 3.9 (range 1–34; 95% CI 3.7–4.2) respectively.

**Table 1:** **Characteristics of included studies and mean number of citations**

	Number (%)	Mean citations (95% CI)
Type of journal		
Local	189 (24.6)	0.29^#^ (0.19–0.39)
International	579 (75.4)	2.12^#^ (1.80–2.44)
Type of study		
Research study	425 (55.3)	2.04^#,β^ (1.67–2.41)
Audits or Editorials or Letters	159 (20.7)	0.88^#^ (0.53–1.23)
Systematic review and/or meta-analysis	62 (8.1)	3.50^#,β^ (2.18–4.82)
Case report or case series	122 (15.9)	0.49^β^ (0.30–0.68)
Research study design		
Case Control studies	45 (10.6)	2.69^#^ (1.54–3.84)
Descriptive Cross-sectional studies	115 (27.1)	1.17^#,β,α^ (0.84–1.49)
Cohort studies	61 (14.4)	2.13 (0.91–3.36)
Experimental studies	71 (16.7)	3.08^β^ (1.81–4.35)
Pre-clinical studies	83 (19.5)	1.18^μ^ (0.67–1.69)
Clinical studies	26 (6.1)	3.73^α,μ^ (2.17–5.30)
Other	24 (5.6)	2.83 (0.34–5.32)
Country of study		
Only in Sri Lanka	639 (83.2)	1.12^#,β^ (0.94–1.29)
Only abroad	104 (13.5)	4.11^#^ (3.02–5.19)
Both in Sri Lanka and abroad	25 (3.3)	5.72^β^ (2.20–9.24)
Type of collaboration		
Local collaboration^*^	115 (30.7)	1.53^#,β,α^ (0.97–2.09)
Local and regional collaborations^*^	47 (12.6)	3.17^#^ (2.67–4.67)
Local and international^*^	184 (49.2)	3.18^β^ (2.49–3.87)
Local, regional and international	28 (7.5)	4.07^α^ (1.28–6.86)

*Local collaboration refers to participation of researchers from multiple institutions in Sri Lanka, Regional collaboration refers to collaborations between South Asian countries and International collaboration refers to collaborations with countries other than South Asian countries; ^α,β,μ,#^ Values under each variable in a single column with the same superscript are significantly different from each other (p < 0.01); CI–Confidence Interval.

The mean number of citations within 2 years of publication was 1.7 (range 0–27; 95% CI 1.4–1.9). Among the publications, 52.1% (n = 400) were never cited within the first two years of publication. Studies published in international journals had a significantly higher number of citations than those published in local journals (Table 1). Research studies and Systematic reviews/Meta analysis carried a higher number of citations than other study types, when considering the study designs of research studies, experimental studies and clinical trials had the highest number of citations (Table 1). Studies conducted abroad or both in Sri Lanka and abroad had a higher number of citations than studies conducted in Sri Lanka alone (Table 1). The mean number of citations for collaborative studies (2.74; 95% CI 2.27–3.21) was significantly higher than that of non-collaborative studies (0.66; 95% CI 0.53–0.79). Regional and international collaborative studies had a significantly higher mean number of citations than solely local collaborations (Table 1). The mean number of citations did not significantly change depending on whether the publication had a positive result (2.08; 95% CI 1.64–2.51) or not (2.92; 95% CI 1.34–4.51) and was also not influenced by the presence (2.30; 95% CI 1.48–3.12) or absence (1.99; 95% CI 1.62–2.36) of the main study conclusion in the title of the article. The number of citations had a significantly positive correlation with the number of authors (*r* = 0.435, p < 0.001), SJR (*r* = 0.354, p < 0.001) and number of words in the title (*r* = 0.187, p < 0.001). However, there was no significant correlation between the number of subjects in the study and the citations (*r* = 0.110, p-NS).

The results of the linear regression analysis using the ‘number of citations’ as the continuous dependent variable and other independent variables are shown in Table 2. The overall model was statistically significant and the R-Square value was 0.301. The journal rank (SJR), number of authors, conducting the study abroad, being a research study or systematic review/meta-analysis and having regional and/or international collaboration all significantly increased the number of citations (Table 2). However, number of words in title and publication of the article in an international journal did not influence citation in the regression analysis.

**Table 2:** **Results of the linear regression analysis**

	Un-standardized β Coeffixcients	P value
Country of study		
Only in Sri Lanka (reference)		
Only abroad	2.20 (1.04–3.36)	<0.001
Both in Sri Lanka and abroad	2.28 (1.24–4.34)	<0.05
Journal Rank (SJR)	1.67 (1.05–2.10)	<0.001
Number of authors	0.42 (0.28–0.56)	<0.001
Number of words in title	0.08 (−0.01–0.17)	NS
Type of journal		
Local (reference)		
International	0.05 (−2.02–2.13)	NS
Type of study		
Case report or case series (reference)		
Research study	1.55 (0.86–2.24)	<0.001
Audits or Editorials or Letters	0.37 (−0.43–1.18)	NS
Systematic review and/or meta-analysis	3.01 (1.96–4.06	<0.001
Collaboration		
Absent (reference)		
Present	0.08 (−0.78–0.22)	NS
Type of Collaboration		
Local only (reference)		
Local and regional	1.64 (0.08–3.19)	<0.05
Local and international	1.65 (0.58–2.72)	<0.01
Local, regional and international	2.54 (0.65–4.44)	<0.01

## Discussion

To our knowledge this is the first comprehensive study evaluating the determinants of citation rates for medical research publications from a developing country. Furthermore previous studies from developed countries have focused only on the citation rates of few specific journals or have been confined to a specific medical sub-speciality (Figg et al. 2006; Kulkarni et al. 2007; Bhandari et al. 2007; Akre et al. 2011; Jacques and Sebire 2010b; Callaham et al. 2002). Citations are a hallmark of academic achievement for authors and for journals, and it correlates highly with the opinion of peers as to a scientist’s contribution to his/her field (Davies et al. 1996). In addition citations are frequently used by medical school deans for promotion reviews (Davies et al. 1996). Our results demonstrate that the journal rank (SJR), number of authors, conducting the study abroad, being a research study or systematic review/meta-analysis and having regional and/or international collaborations all significantly increased the number of citations.

Among the Sri Lankan medical research publications between years 1997–2006, 52.1% were never cited within the first two years of publication. A significantly higher proportion when compared with other similar studies, where approximately only 20% of the articles were never cited (Schwartz 1997; Callaham et al. 2002). This could partly be explained by the fact that the Ceylon Medical Journal had the highest proportion (22.9%) of the publications, and that 58.5% of the journals that carried publications by Sri Lankan authors had a SJR <0.5. The SJR for the Ceylon Medical Journal during the period concerned was between 0.11–0.14, which is comparatively much lower than that of other high impact medical journals such as the British Medical Journal (SJR 2.1–2.8) and the Lancet (SJR 2.2–4.3). In addition the SJR of the publishing journal was a strong predictor of citations. Previous studies have also demonstrated similar results, a review of emergency medicine papers by Callaham et al. revealed that the journal impact factor was associated with the highest increase in the citation rates (Callaham et al. 2002). The journal impact factor or SJR may be viewed by citing authors as an indicator of the quality of the published article. Therefore, a strong or decisive paper submitted to a low impact journal might not receive the full scientific recognition it deserves and vice versa (Callaham et al. 2002).

Studies have shown that there is a severe under representation of biomedical research from developing countries in high impact medical journals, only 6.5% of the publications in these journals have authors from developing countries where 90% of the world’s population lives (Sumathipala et al. 2004). Reasons for such under representation include barriers such as inadequate funding, facilities, technical support and training. One effective method to overcome such barriers is by inter- and intra-regional scientific collaborations to solve common health issues. Collaboration is a means to spread expenses over different individuals and institutions, enhance intellectual synergy, and allow resources to be shared (Figg et al. 2006). Our results clearly demonstrate that collaborations and number of authors (an indirect measure of collaboration) increases the number of citations, with the highest increases in the citation rate observed in studies with local, regional and international collaboration. Previous studies have also shown that investigators who are open to collaborations produce a superior research product that results in a higher impact as measured by number of citations (Figg et al. 2006). In addition collaborations also increased the possibility of publication in a higher impact journal, the mean SJR for journals increased significantly with the increasing level of collaborations (no collaboration–0.41; local only–0.56; local and regional–0.90; local and international–1.15; local, regional and international–1.58).

The type of the publication also had a significant influence on citations. At present there is a widely accepted hierarchy of evidence amongst the scientific community, based on the relative reliability of various types of study designs (Harbour and Miller 2001). The citation impact of various study designs in the present study generally followed this hierarchical order of evidence. Being a systematic reviews and/or meta-analysis had the most influence on the number of citations. In the different research study designs, clinical trials and experimental studies had a higher number of citations, with descriptive cross-sectional studies having the lowest mean citation rate. Interestingly the presence of a positive result in the study did not influence the citation rate, and the journal SJR did not vary significantly between studies with and without a positive result. These findings are in keeping with results from previous similar studies (Callaham et al. 2002). However, it is in contrast to the consistent bias by journals towards the acceptance and publication of studies with positive results (Easterbrook et al. 1991; Johnson and Dickersin 2007). We did not observe a significant relationship between the length of the article title and the presence of research conclusion in the title, with the subsequent citation rate in the multivariate analysis. However Jacques and Sebire, observed that the construction of an article title had a significant impact on the citation rate. These findings have been contradicted by other authors, who have shown that brief and concise titles are associated with higher citation counts (Paiva et al. 2012). Longer titles seem to be associated with higher citation rates mostly in journals with high impact factors (Habibzadeh and Yadollahie 2010). This might partly explain our observation, since majority of Sri Lankan research publications were in low impact journals.

There are several limitations that need to be taken in to account when drawing conclusions from our findings. We did not assess the quality of the citation or whether it supported the index paper. A citation may not credit or praise a study but instead refute or criticize it. In addition citation rates do not necessarily translate into clinical or scientific impact, but since true impact is extremely difficult to measure, we are compelled to use citations as a surrogate marker. Furthermore, our results relates to only one developing country, however most developing countries share common health care issues and barriers to research. Allowing for this and other limitations, our evaluation provides empirical evidence on the factors determining citation rates for publications by authors from a developing country.

## Conclusions

The total number of medical research publications from Sri Lanka has steadily increased from 1997 to 2006; however absolute numbers still remain relatively low. A significant majority of research papers are never cited indicating poor dissemination of knowledge. The journal rank, number of authors, conducting the study abroad, being a research study or systematic review/meta-analysis and having regional and/or international collaboration all significantly increased the number of citations. However, the presence of a positive result in the study did not influence the citation rate. Inter- and intra-regional scientific collaborations to solve common health issues will help in tackling barriers to research and also increase citation rates.

## References

[CR1] Akre O, Barone-Adesi F, Pettersson A, Pearce N, Merletti F, Richiardi L (2011). Differences in citation rates by country of origin for papers published in top-ranked medical journals: do they reflect inequalities in access to publication?. J Epidemiol Community Health.

[CR2] Amin M, Mabe MA (2003). Impact factors: use and abuse. Medicina.

[CR3] Bhandari M, Busse J, Devereaux PJ, Montori VM, Swiontkowski M, Tornetta Iii P, Einhorn TA, Khera V, Schemitsch EH (2007). Factors associated with citation rates in the orthopedic literature. Can J Surg.

[CR4] Callaham M, Wears RL, Weber E (2002). Journal prestige, publication bias, and other characteristics associated with citation of published studies in peer-reviewed journals. JAMA.

[CR5] Commission on Health Research for Development (1990). Health research: essential link to equity in development.

[CR6] Davies HD, Langley JM, Speert DP (1996). Rating authors’ contributions to collaborative research: the PICNIC survey of university departments of pediatrics. Pediatric Investigators’ Collaborative Network on Infections in Canada. CMAJ.

[CR7] Department of Census and Statistics Sri Lanka (2010). Statistical pocket book [online].

[CR8] Druss BG, Marcus SC (2005). Growth and decentralization of the medical literature: implications for evidence-based medicine. J Med Libr Assoc.

[CR9] Easterbrook PJ, Berlin JA, Gopalan R, Matthews DR (1991). Publication bias in clinical research. Lancet.

[CR10] Falagas ME, Kouranos VD, Arencibia-Jorge R, Karageorgopoulos DE (2008). Comparison of SCImago journal rank indicator with journal impact factor. FASEB J.

[CR11] Figg WD, Dunn L, Liewehr DJ, Steinberg SM, Thurman PW, Barrett JC, Birkinshaw J (2006). Scientific collaboration results in higher citation rates of published articles. Pharmacotherapy.

[CR12] Habibzadeh F, Yadollahie M (2010). Are shorter article titles more attractive for citations? Cross-sectional study of 22 scientific journals. Croat Med J.

[CR13] Hamilton DP (1991). Research papers: who’s uncited now?. Science.

[CR14] Harbour R, Miller J (2001). A new system for grading recommendations in evidence based guidelines. BMJ.

[CR15] Jacques TS, Sebire NJ (2010). The impact of article titles on citation hits: an analysis of general and specialist medical journals. JRSM Short Rep.

[CR16] Jacques TS, Sebire NJ (2010). The impact of article titles on citation hits: an analysis of general and specialist medical journals. JRSM Short Rep.

[CR17] Johnson RT, Dickersin K (2007). Publication bias against negative results from clinical trials: three of the seven deadly sins. Nat Clin Pract Neurol.

[CR18] Kulkarni AV, Busse JW, Shams I (2007). Characteristics associated with citation rate of the medical literature. PLoS One.

[CR19] Paiva CE, Lima JP, Paiva BS (2012). Articles with short titles describing the results are cited more often. Clinics.

[CR20] Ranasinghe P, Perera Y, Abeygunasekera A (2011). The process and costs of publishing medical journals in Sri Lanka: an economic evaluation. BMJ Open.

[CR21] Ranasinghe P, Jayawardena R, Katulanda P (2012). Sri Lanka in global medical research: a scientific analysis of the Sri Lankan research output during 2000-2009. BMC Res Notes.

[CR22] Schwartz CT (1997). The rise and fall of uncitedness. Coll Res Libr.

[CR23] Seglen PO (1997). Citations and journal impact factors: questionable indicators of research quality. Allergy.

[CR24] Sri Lanka Department of Health Service (2002). Annual health bulletin.

[CR25] Stringer MJ, Sales-Pardo M, Nunes Amaral LA (2008). Effectiveness of journal ranking schemes as a tool for locating information. PLoS One.

[CR26] Sumathipala A, Siribaddana S, Patel V (2004). Under-representation of developing countries in the research literature: ethical issues arising from a survey of five leading medical journals. BMC Med Ethics.

[CR27] Willis DL, Bahler CD, Neuberger MM, Dahm P (2011). Predictors of citations in the urological literature. BJU Int.

